# Challenges and Strategies for Proteome Analysis of the Interaction of Human Pathogenic Fungi with Host Immune Cells

**DOI:** 10.3390/proteomes3040467

**Published:** 2015-12-14

**Authors:** Thomas Krüger, Ting Luo, Hella Schmidt, Iordana Shopova, Olaf Kniemeyer

**Affiliations:** 1 Department of Molecular and Applied Microbiology, Leibniz Institute for Natural Product Research and Infection Biology, Hans Knöll Institute (HKI), Adolf-Reichwein-Str. 23, 07745 Jena, Germany; E-Mails: thomas.krueger@leibniz-hki.de (T.K.); ting.luo@leibniz-hki.de (T.L.); hella.schmidt@leibniz-hki.de (H.S.); iordana.shopova@leibniz-hki.de (I.S.); 2 Institute of Microbiology, Friedrich Schiller University Jena, Adolf-Reichwein-Str. 23, 07745 Jena, Germany

**Keywords:** host-pathogen interaction, *Aspergillus fumigatus*, *Candida albicans*, fungal infections, mass spectrometry, immunoproteomics, neutrophils, macrophages, phagolysosome

## Abstract

Opportunistic human pathogenic fungi including the saprotrophic mold *Aspergillus fumigatus* and the human commensal *Candida albicans* can cause severe fungal infections in immunocompromised or critically ill patients. The first line of defense against opportunistic fungal pathogens is the innate immune system. Phagocytes such as macrophages, neutrophils and dendritic cells are an important pillar of the innate immune response and have evolved versatile defense strategies against microbial pathogens. On the other hand, human-pathogenic fungi have sophisticated virulence strategies to counteract the innate immune defense. In this context, proteomic approaches can provide deeper insights into the molecular mechanisms of the interaction of host immune cells with fungal pathogens. This is crucial for the identification of both diagnostic biomarkers for fungal infections and therapeutic targets. Studying host-fungal interactions at the protein level is a challenging endeavor, yet there are few studies that have been undertaken. This review draws attention to proteomic techniques and their application to fungal pathogens and to challenges, difficulties, and limitations that may arise in the course of simultaneous dual proteome analysis of host immune cells interacting with diverse morphotypes of fungal pathogens. On this basis, we discuss strategies to overcome these multifaceted experimental and analytical challenges including the viability of immune cells during co-cultivation, the increased and heterogeneous protein complexity of the host proteome dynamically interacting with the fungal proteome, and the demands on normalization strategies in terms of relative quantitative proteome analysis.

## 1. Introduction

Superficial, non-life-threatening infections of the human skin, nails and mucosa are the most common fungal diseases in humans and affect around one quarter of the world population., Infectious diseases caused by fungi also contribute substantially to human morbidity and mortality. In particular, invasive fungal infections are associated with high mortality rates, which often exceed 50%. Altogether, 1.5 million people are estimated to be killed by invasive mycoses worldwide each year. More than 90% of these deaths are caused by fungi of the four genera: *Candida*, *Aspergillus*, *Cryptococcus*, and *Pneumocystis* [1]. Immunocompromised patients are particularly vulnerable to these fungal killers, whereas invasive fungal infections are extremely rare in immunocompetent individuals [2].

*Candida* species are a polyphyletic group, which is part of the commensal flora of the gastrointestinal tract in more than a half of the healthy population [2]. Under certain conditions, *Candida* species are capable of causing a range of infections from superficial to dangerous invasive infections, designated as invasive candidiasis. Systemic *Candida* infections have a high clinical relevance: They account for more than 70% of all invasive fungal infections in immunocompromised and critically ill patients [3] and cause 8% of all nosocomial blood stream infections in the United States [4]. Worldwide, *Candida albicans* remains the most frequently isolated agent of candidiasis, but non-*Candida albicans* species have gained clinical importance [5]. *Candida albicans* is undoubtedly the best studied pathogenic *Candida* species and several virulence traits have been identified so far. Among them are their ability to grow in the yeast or hyphal form (dimorphism), the production of molecules, which mediate adhesion and invasion, the formation of biofilms, the secretion of hydrolases, and the acquisition of essential trace metals [6].

In contrast to *Candida*, filamentous fungi of the genus *Aspergillus* are soil-borne fungi with a saprophytic life style [7]. Their asexually produced spores are easily dispersed into the air and due to their small diameter they penetrate deep into the respiratory tract upon inhalation. Because of that, most invasive *Aspergillus* infections disseminate from the lungs [8]. Patients at risk for developing invasive aspergillosis include neutropenic and critically ill patients as well as patients on high-dose steroid therapy [9]. *A. fumigatus* is the major cause of invasive aspergillosis in transplant patients (65%) followed by *A. flavus* and *A. niger* [10]. Exposure to *Aspergillus* conidia can also lead to chronic infections and allergic responses, which result in allergic bronchopulmonary aspergillosis (ABPA) and severe asthma [9]. Due to its medical importance, the virulence traits of *A. fumigatus* have been most intensively studied and are based on multiple factors. The acquisition of iron by siderophores and the defense against immune effector cells based on the pigment 1,8-dihydroxynaphtalene melanin are the most prominent examples [11,12].

The basidiomycetous yeast *Cryptococcus* is more distantly related to the genera *Candida* and *Aspergillus*. *Cryptococcus* infections occur by the inhalation of infectious cells and are considered a primary pulmonary illness. Nevertheless, disseminated infections often lead to inflammatory diseases of the central nervous system [13]. Among the 37 recognized species of *Cryptococcus*, *C. neoformans*, and *C. gattii* are the major pathogens to humans. *C. neoformans* infections occur mostly in immunodeficient individuals, particularly in patients with AIDS. *C. gattii* can also infect immunocompetent hosts and has traditionally been considered as “tropical” or “subtropical” fungus”. Despite that, endemic outbreaks were reported from Vancouver Island, Canada [14]. The polysaccharide capsule is the major virulence factor of *Cryptococcus* to evade host defenses [15], but also the formation of melanin and urease activity function as virulence determinants [16,17].

Pneumonia caused by the opportunistic pathogenic fungus *Pneumocystis jirovecii* is the most prevalent opportunistic infection in patients with AIDS. It causes little or no disease in healthy individuals. The fungus is most probably transmitted via aerosols from person-to-person and exists almost exclusively within the alveoli of the lung and does not invade the host cell. Since Pneumocystis species have not yet been isolated in pure culture, little is known about their biology and pathogenicity determinants [18,19].

In addition to the aforementioned fungi, several other species are able to cause severe diseases in humans. Their occurrence is either restricted to a specific region of the world or the frequency of infections is relatively rare.Nonetheless, mucormycosis has emerged as the third most common invasive infection after candidiasis and aspergillosis in patients with hematological malignancies and allogeneic stem cell transplantation. Mucormycosis is caused by filamentous fungi of the order Mucorales in the class Zygomycota. Medically most significant are species of the genera *Rhizopus*, *Lichtheimia*, and *Mucor* [20].

Another group of ascomycetes are termed the dimorphic fungal pathogens. They cause diseases in endemic regions of the world and include *Histoplasma capsulatum*, *Blastomyces dermatidis*, *Coccidioides immitis*, *Paracoccidioides brasiliensis*, *Sporothrix schenkii*, and *Penicillium marneffei*. A common feature of these species is that they grow as molds in soil at ambient temperature and convert to pathogenic yeasts after infectious spores are inhaled by humans [21,22].

The interplay between the fungal pathogen and the human host is only partially understood. It is evident that the human professional phagocyte population consisting of monocyte/macrophages, polymorphonuclear leukocytes (neutrophils/PMNs) and dendritic cells (DCs) plays a central role in the defense against fungi. Usually, macrophages are the first cells to encounter an invading fungus. They recognize fungal pathogens via pathogen-associated molecular patterns (PAMPs), phagocytose, and consecutively kill them intracellularly. In addition, they generate a proinflammatory response to activate further immune cells. Neutrophils are the most abundant phagocyte population, which is immediately recruited to the sites of infection. They have high phagocytic activity and are endowed with powerful oxidative and non-oxidative microbicidal components [23,24,25]. Besides phagocytosis, neutrophils possess an array of extracellular killing mechanisms including the formation of neutrophil extracellular traps (NETs). NETs are characterized by the release of extracellular DNA associated with histones and granular and cytoplasmic proteins, which exhibit antimicrobial activity [26,27,28]. In contrast, dendritic cells (DCs) are important antigen-presenting cells that act as messengers between the innate and adaptive immune system. They have been shown to be important for the discrimination between different fungal morphotypes or growth stages [29].

Little is known about the contribution of the adaptive immune system to confer resistance against fungal pathogens. It is generally accepted that the development of a specific T_h_ response contributes to the susceptibility to invasive mycoses. In contrast, there is a lack of clear evidence that antibodies confer protection against pathogenic fungi [30].

In short, fungal infections are controlled primarily by the host innate immune system. Knowledge about the interplay between fungal pathogens and immune cells has increased recently due to the investigation of host-pathogen interaction transcriptomes [31,32,33,34,35,36,37]. Transcriptomic profiles of the interaction of pathogenic fungi with epithelial or endothelial cells have also been examined [38,39,40,41,42]. Due to technical challenges such as sample quantity, complexity, and heterogeneity, proteomic studies on this topic are still rare. Several proteomic data are available from *C. albicans*, but two of the few examples from *A. fumigatus* described the response of human bronchial epithelial cells and endothelial cells in response to this pathogenic mold [43,44]. Here, we review current efforts and strategies to investigate the proteomic changes during interaction of pathogenic fungi with immune effector cells in the human host. We also give a brief overview about the investigation of fungal-specific serum antibody signatures in patients with invasive mycoses.

## 2. Immunoproteomics

In clinical fungal infection studies, circulating serum antibodies are important molecular markers as they reflect a molecular imprint of antigens of infectious agents. In addition, antigens specific for certain fungal pathogens are promising candidates for diagnostic biomarkers and vaccination strategies.

The first proteomics study on immunoreactive protein antigens of a pathogen interacting with the host humoral immune response was reported for *Borrelia burgdorferi by* Jungblut in 1999 [45]. Later, in 2001, the term “immunoproteomics” arose to define studies on large sets of proteins involved in the humoral immune response [46]. Over the years, the technical advances in the field of proteomics have markedly facilitated the detection of pathogen-specific antigens.

### 2.1. Gel-Based Immunoproteomics

The combinatorial approach of 2D-GE followed by immunoblotting is highly effective to isolate and identify antigenic proteins (immunoproteome). This approach has been defined as serological proteome analysis (SERPA) [47]. The principle works as follows: Immunoreactive proteins are two-dimensionally separated, transferred onto a membrane, and probed with patient serum, which presumably contains certain pathogen-related antibodies. Although antigens are denatured by 2D-GE and only linear epitopes can be detected, post-translational protein modifications that could be part of epitopes and affect antigen-antibody recognition are still retained during the denaturation step.

In the last decade, several serological proteome analyses with focus on antigens of human pathogenic fungi have been conducted. Pitarch and co-workers decoded the serological responses of the host to the cell wall proteome as well as the intracellular proteome of *C. albicans* to identify novel diagnostic, prognostic, and therapeutic candidate markers for systemic candidiasis [48,49,50]. Although *C. albicans* is a commensal in the human gut provoking a basic and persistent anti-Candida antibody level in the host, the authors found that a pattern of 22 IgG serum antibodies (mainly against glycolytic enzymes and heat shock proteins) can differentiate invasive candidiasis (IC) from non-IC patients by using unsupervised clustering analyses. The authors highlighted that the serum IgG antibody signature directed against heat shock protein 90 (Hsp90) and enolase 1 (Eno1) of *C. albicans* can be applied for IC diagnosis in non-neutropenic patients. Later, the same group combined fingerprints of IgG antibodies to two distinct protein species of Eno1 and Pfk1 (phosphoglycerate kinase) to discriminate candidemia from non-infected patients [51]. Similar studies have also been carried out for *Aspergillus fumigatus*. Due to the allergy invoking capacity of *A. fumigatus*, many studies focused on screening for immunoreactive anti-*Aspergillus* IgE antibodies. Glaser *et al.* [52] detected specific IgE antibodies against the phialide cell wall protein PhiA in the sera of 94% of all investigated ABPA patients. This protein was identified as a major allergen and may be regarded as a potential tool for specific diagnosis of allergic sensitization against *A. fumigatus*. The serological response to *A. fumigatus* protein antigens in patients with invasive aspergillosis has been investigated as well [12]. Even antibodies specific to an enzyme involved in the biosynthesis of the mycotoxin gliotoxin were proposed as a potential biomarker for the diagnosis of IA in non-neutropenic patients [53].

Overall, the SERPA approach has a high resolution in protein separation and certain post-translational modifications of antigens remain retained, which can be visualized by suitable gel staining methods. However, this workflow is very time-consuming and requires great skill of the operator to ensure reproducibility. Moreover, only the most abundant and soluble proteins can be sufficiently resolved on the immunoblot and multiplexing [e.g., as applied for the difference gel electrophoresis technique (DIGE)] of different conditions, genotypes, and culturing time points is excluded for sera screening.

### 2.2. Gel-Free Immunoproteomics

The protein array is another high-throughput technology, which is applied for immunoproteomic studies. Complex protein samples from cells or tissues can be fractionated by multiple LC steps based on protein pI or hydrophobicity. A variety of technologies are available to spot protein sub-fractions onto the planar surfaces in ordered arrays [54,55,56]. By applying patient antibodies to the protein arrays, protein antigen fractions are detected with the help of secondary labeled antibodies., After localization of interesting antigens on the array, the reactive antigen from the selected protein fraction has to be isolated and identified by further fractionation, immunoprecipitation, and MS detection. Instead of using protein sub-fractions, expressed recombinant proteins or peptides can also be used in this approach to produce protein arrays or multiplex bead arrays. Mochon *et al.* [57] reported on a *C. albicans* protein microarray used for comparison of serological profiling of *C. albicans* in different stages of candidemia. The authors selected a set of cell surface proteins according to the *Candida* Genome Database (CGD, http://www.candidagenome.org/) and expressed interesting candidates in *E. coli*. Despite the fact that the immunocompetent host exists in a permanent host-pathogen interplay with the commensal *C. albicans*, a set of 13 cell surface antigens mainly involved in either oxidative stress or drug resistance were identified [57]. These candidates were specific for acute candidemia. Due to the cell free nature of *in vitro* translated peptides, potential epitopes could get lost due to protein misfolding or a lack of post-translational modifications (e.g., glycosylations), which may affect the conformational structure of the native protein and its binding affinity.

Immunocapture MS is referred to as inverse immunoproteomics, since patient antibodies are firstly immobilized on the protein array to investigate antigen profiles. This approach is highly efficient allowing the simultaneous processing of large numbers of patient samples and an easy handling of native antigens in solution. Furthermore, low molecular weight (LMW) antigens (<20 kD) are more sensitively detected by this approach [58].

Altogether, the SERPA approach and protein microarrays bring both advantages and disadvantages. SERPA requires less prior knowledge and is the ideal choice for the identification of potentially interesting fungal protein antigens. Protein/peptide microarrays, on the other hand, are more suitable for high-throughput screenings of serum samples and the generation of quantitative data. The combination of both methods has the highest potential for the diagnosis and immunotherapy of invasive fungal infections. In addition, LC-MS/MS-based approaches allow the identification of peptides presented on major histocompatibility complexes (MHCs) on the cell surface of immune cells.

## 3. Interaction of Macrophages with Human Pathogenic Fungi

Macrophages play a major role in both the innate immune response against invading pathogens and the initiation of the adaptive immunity by recruitment of other immune cells, especially lymphocytes. Furthermore, macrophages can regulate the inflammatory response of the host [59]. The distinct importance of macrophages is notably evident bearing in mind that these phagocytic cells are found in essentially all tissues [60]. In the following section, an overview about the interplay of macrophages with human pathogenic fungi will be outlined, followed by an overview about recent proteomic studies on host-pathogen interactions.

### 3.1. Recognition of the Fungal Pathogen

The uptake of pathogens by macrophages is mediated by receptor activation. Receptors either bind antibodies or complement factors deposited on the surface of the fungal pathogen (Fc and complement receptors), or pattern recognition receptors (PRR) perceive distinct PAMPs) on the fungal surface. The most important fungal PAMPs are the cell wall carbohydrates β-glucan and chitin as well as mannoproteins [61]. To avoid detection, immunostimulatory PAMPs are often shielded by the fungal pathogen, e.g., resting conidia of *A. fumigatus* are coated with melanin and the hydrophobic protein RodA, so that they are immunologically inert [62,63,64]. Swelling of the conidia leads to the sequential loss of the protective layer and exposure of β-1,3-glucans, galactomannans and chitins that are recognized by macrophage receptors [65,66,67]. In yeast cells of *C. albicans*, the β-glucan layer is obscured by outer mannoproteins [68], whereas in *C. neoformans* the polysaccharide capsule and the secretion of an antiphagocytic protein inhibit phagocytosis [69].

The polysaccharide β-1,3-glucan is a major component of the fungal cell wall [70,71]. Its binding to the C-type lectin Dectin-1 receptor triggers phagocytosis and initiates cytokine and ROS production [72,73]. Further innate receptors, such as the Toll-like receptors TLR 2 (CD282), 4 (CD284), and 9 (CD289) as well as DC-SIGN (dendritic cell-specific intercellular adhesion molecule-3-grabbing non-integrin, CD209) are able to differentiate between variable morphological states of fungal pathogens and modulate the host immune response [74,75,76,77,78,79,80].

### 3.2. Phagocytosis, Phagosomal Maturation and Killing of the Pathogen

Phagocytosis requires reorganization of the actin cytoskeleton and extension of the plasma membrane, which is regulated by signaling [81,82]. Downstream activation leads to the internalization of fungal cells within a membrane -enclosed intracellular organelle, the phagosome. The phagosomal membrane is derived from different sources such as the plasma membrane, de novo synthesis and intracellular organelles like the endosomes, lysosomes, and the ER [83]. The newly formed phagosome fuses with lysosomes and endocytic vesicles to finally mature into a biocidal phagolysosome that kills the fungal pathogen. Maturation is accompanied with the acquisition of a specific set of marker proteins indicative for the maturation stage of the phagolysosome [82]. Maturation involves subsequent acidification of the phagolyososomal lumen driven by the vacuolar proton pump (vATPase) [64]. It also involves assembly and activity of the NADPH oxidase to generate reactive oxygen and nitrogen species (ROS, RNS) [84]. Further recruited proteins have functions in intracellular signaling, trafficking, and vesicle fusion and regulate the maturation process. An efficient killing of the pathogen is accomplished by the joint effects of acidification, ROS/RNS production and activity of hydrolytic enzymes of lysosomal origin.

Human pathogenic fungi have evolved ingenious strategies to subvert the lysosomal system, such as the inhibition of the fusion of the phagosome membrane enriched in lysobisphosphatidic acid and the V-ATPase required to lower the pH in the phagosomal lumen. Besides this early counter defense mechanism, the capability to induce an active recycling of the lysosome-associated membrane glycoprotein 1 (LAMP-1) and the lysosomal protease cathepsin D out of the phagosomes has been reported for *C. albicans*. Furthermore, an active suppression of the nitric oxide (NO) production in macrophages has also been observed for *C. albicans* [85].

### 3.3. Studying the Phagolysosomal Proteome

A few proteomics studies have been published so far dealing with the immune response of macrophages against human pathogenic fungi. By far, more data are available from viral [86,87,88,89,90] and bacterial [91,92,93,94,95] pathogens.

With regard to fungi, proteomic analyses of the interaction of pathogenic fungi with macrophages have mainly been studied in *C. albicans* [96,97,98,99,100]. The first proteomics study on *C. albicans*-macrophage interaction was performed by Fernández-Arenas *et al.* [99]. The authors developed an *in vitro* model of phagocytosis to discriminate between internalized and attached but non-ingested yeast cells utilizing a differential staining procedure based on a pre-labeling with Oregon Green 488 dye and a counterstaining with calcofluor white following the incubation. This provided the basis to establish an enrichment protocol for ingested yeasts that used a Triton X-114 solution to lyse the macrophage cells and to keep the *C. albicans* yeast cells intact. The following proteomics analysis by 2D-GE and MS identified 132 differentially expressed yeast proteins. A rapid protein response of the fungus was observed in terms of a metabolic shift to a starvation mode and an activation of the degradation and detoxification protein machinery [99]. In this context, Carneiro *et al.* [101] reported a sophisticated fluorescence staining method that allows accurate quantification of living from dead phagocytes as well as internalized from non-internalized cells, in which yeast fluorescence remains maintained within phagocytes even under acidifying conditions. The method is based on the quenching effect of propidium iodide over Sytox-Green fluorescence to assess yeast phagocytosis by flow cytometry [101].

More proteome studies have been performed on the host side. In most cases, no sophisticated cell separation methods were applied and non-phagocytosed fungal cells were simply removed by several washing steps and macrophage cells were subsequently lysed in a protein extraction buffer.

By studying the response of murine macrophages to both heat-inactivated and live *C. albicans*, several differentially regulated proteins have been observed (2D-GE, MALDI-TOF) that are involved in cytoskeletal organization, signal transduction, metabolism, protein biosynthesis, stress response, and protein fate. In contrast to live cells, heat-inactivated *C. albicans* showed an anti-inflammatory response and significantly (*p* < 0.05) decreased TNF-α secretion as well as lower ERK1/2 phosphorylation levels [97]. Similar data about the alteration of protein expression in macrophages were obtained by Shin *et al.* [102]. They reported a downregulation of the energy metabolism in macrophages, which was illustrated by lower levels of key glucose metabolic enzymes and proteins involved in protein synthesis upon *C. albicans* infection. In another study, the response of murine macrophages to *C. albicans* SC5314 was analyzed by a subcellular fractionation procedure (cytosol, organelle/membrane and nucleus enriched fractions) to cover low abundant proteins with 2D-GE. The sub-proteomic fractions were labeled with fluorescent dyes to perform relative quantification based on the DIGE technique. Differentially regulated proteins during the interaction of macrophages were involved in immune, pro-inflammatory and oxidative responses, the unfolded protein response, and apoptosis. Interestingly, for the PRR Galectin-3, not only an increase in abundance, but also a distinct allocation along the interaction was observed, namely more Galectin-1 was found extracellularly [98]. Later, the same group investigated the phosphoproteome of RAW 264.7 macrophages in response to co-incubation with *C. albicans*. The most striking result was a prevalence of anti-apoptotic markers during interaction with the yeast. This suggests an inhibition of apoptosis by *C. albicans* [103]. A further example for macrophage reprogramming by *C. albicans* was shown in a comparative proteomic study with human pro-inflammatory M1 macrophages and anti-inflammatory M2 macrophages [104]. An M1-to-M2 switch in polarization was observed in response to *C. albicans*, which may increase *C. albicans* survival in the human host.

In summary, proteomic studies clearly showed that *C. albicans* cells phagocytosed by macrophages switch to a starvation-like response, but at the same time they show immunomodulatory activity by activating an inflammatory response and by inhibiting apoptosis.

### 3.4. Current Methods for the Purification of Phagolysosomes

**Figure 1:**
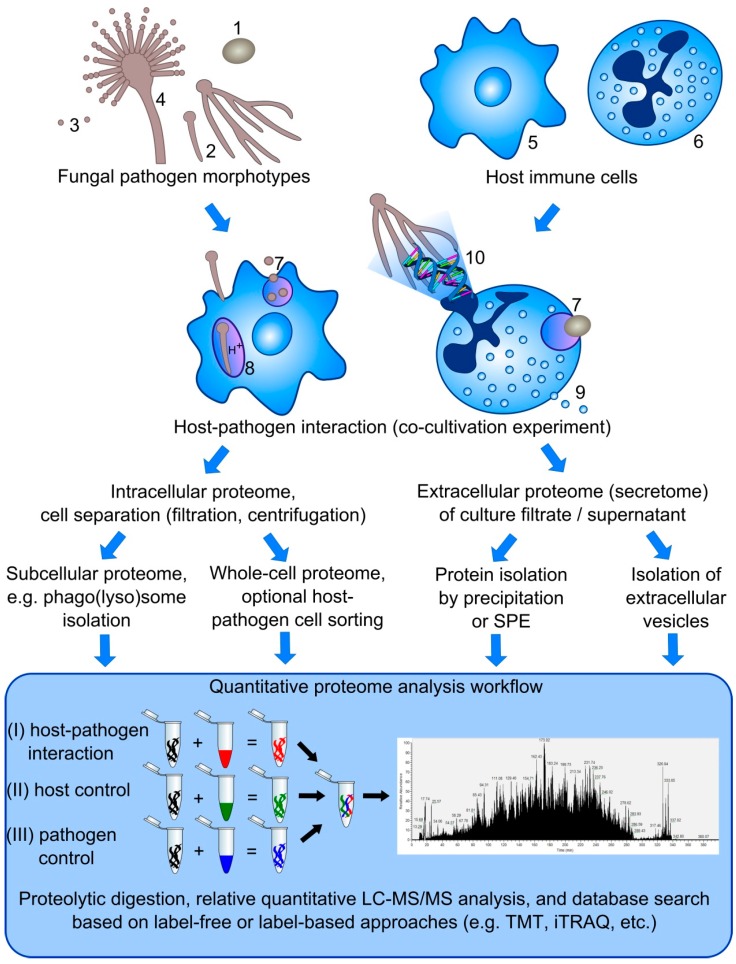
Overview on proteome analysis strategies to study host-pathogen interactions. The interaction of different morphotypes of fungal pathogens, including yeasts (1), hyphae (2), conidia (3), and conidiophore (4) with host immune cells, such as macrophages (5), neutrophils (6), and dendritic cells (not shown), can be studied based on different defense mechanisms, e.g., phagocytosis (7) and phagolysosome maturation (8), degranulation (9), and NET formation (10). Based on the mechanism of interest, several strategies can be applied to investigate the intracellular as well as extracellular proteome (secretome) using label-free or label-based approaches to calculate changes on the protein level in comparison to either host or pathogen control samples.

No proteomic studies on fungal cell-containing phagolysosomes have been published yet. The main bottleneck of proteome analyses of phagolysosomes is to obtain a sufficient amount of proteins after isolation. The first challenge is to disrupt the outer membrane of the macrophages and at the same time enrich intact fungal cell-containing phagolysosomes. As a consequence, proteomic studies on the phagosomal maturation and properties of the organelle require an elaborate protocol for the isolation and purification of phagolysosomes from immune cells (Figure 1). A common way to study the general protein composition of the phagolysosome is to expose macrophages with latex beads. The floating properties of the beads allow the separation of the phagolysosomes from a homogenate by ultracentrifugation on a sucrose gradient [105]. The isolation of pathogen-containing phagolysosomes requires adaptation of the protocol to the individual properties of the relevant pathogen containing phagolysosomes. Altered densities of the pathogen-phagolysosomes, for instance, may hamper a clean purification of the phagolysosome fraction from the cell homogenate or may not yield in satisfying amounts. Lee and co-workers described a purification method based on a sucrose gradient centrifugation to isolate *Mycobacterium bovis* bacilli Calmette-Guérin-containing phagolysosomes (BCG-phagolysosomes) from cell homogenates. They labeled the bacterium with a fluorophore to detect the BCG-phagolysosome positive layer [106].

Alternatively, Urwyler *et al.* [107] and Hofmann *et al.* [108] applied antibody-coupled magnetic beads to isolate and analyze *Legionella pneumophilia* containing vacuoles from immune cells. They identified novel endosomal markers specific for the *Legionella*-host interaction. Upon ingestion by a phagocyte, the intracellular pathogen expresses a range of effector proteins such as the Icm/Dot transporter, which localizes to the phagolysosomal membrane and can be targeted with antibodies coupled to magnetic beads. Subsequent centrifugation on a histogradient allows purifying a clean fraction with *L. pneumophilia* containing vacuoles. Drawbacks of this method are relatively low sample yields and the risk of losses due to the instable composition of the vacuole. Homogenization of the cells needs to be performed carefully to release the phagolysosomes from the cells without breaking the organelle membranes.

To isolate and analyze the proteome of *Mycobacterium-*containing phagolysosomes, Steinhäuser *et al.* [109] established a protocol based on magnetic labeling of the pathogen itself. Cell debris of the homogenate of macrophages with ingested *Mycobacterium* has been pelleted by centrifugation at low speed, so that the pathogen-containing phagosomes remain floating. The supernatant has been applied on a strong magnet to separate the magnetic fraction of pathogen-containing phagolysosomes from the non-magnetic cell debris. The protocol requires an efficient labeling of the bacterium with magnetic beads. Thus, pathogens with hydrophobic surface coatings, such as *A. fumigatus* conidia, are not as easy to label due to hidden linkers on the cell surface. Also, different sedimentation characteristics influence the purity of the fraction which contains pathogen-containing phagolysosomes.

### 3.5. Extracellular Vesicles of Macrophages

Activated macrophages release extracellular vesicles that open further possibilities for investigations in terms of host-pathogen interactions (Figure 1). Cypryk *et al.* [110] analyzed the proteome of extracellular vesicles released from β-glucan-activated macrophages. In this study, 540 vesicular proteins were identified, including several receptors (e.g., cation-dependent mannose-6-phosphate receptor, macrophage scavenger receptor, and P2X7 receptor) and highly abundant integrins as well as their cytoplasmic cargo proteins [110].

## 4. Interaction of Human Pathogenic Fungi with Neutrophils

Human polymorphonuclear granulocytes possess a central indispensable role in orchestrating the innate antifungal response. Increasing experimental evidence from murine models indicates the non-redundant functions of neutrophil effector cells at early stages of invasive fungal infections compared to other important immune cell types [111,112,113,114]. Further evidence is that invasive mold infections occur mainly in neutropenic individuals and chronic granulomatous disease (CGD) patients, whose neutrophils lack the capability of producing oxidative burst [115]. Neutrophils possess several sophisticated defense strategies against fungal pathogens. These include phagocytosis of yeasts, spores, or small spore germlings, extracellular killing of pathogens via reactive oxygen and nitrogen species (ROS/RNS), degranulation of antimicrobial chemokines, and NETs formation [12,27,116].

### 4.1. Neutrophil Phagocytosis and Phagosome Maturation

Macrophages and neutrophils are equipped with a similar set of PRRs that are involved in fungal pathogen recognition. However, neutrophil phagosome maturation differs significantly from the process in macrophages [117]. Unlike macrophages, neutrophil phagosome formation does not follow the canonical endosomal maturation pathway and is not accompanied by phagolysosome acidification. Instead, neutrophils contain azurophilic granules that are rich in anti-microbial defensins and fuse with phagocytic vacuoles in the course of NADPH oxidase complex activation [118,119]. Studies on neutrophil phagosome formation against human pathogenic fungi are quite limited and mainly rely on data obtained via microscopic studies and ROS formation assays. The most recently discovered extracellular strategy of neutrophils to fight invading pathogens is the formation of NETs, which will be discussed in the following section.

### 4.2. Neutrophil Extracellular Traps (NETs)

NETs are networks of extracellular chromatin structures composed of DNA, histones, and antimicrobial peptides, which are released by neutrophils in order to immobilize the pathogen and inhibit its dissemination. Although NETs can be formed against all morphotypes of *A. fumigatus* and *C. albicans*, hyphae are known to trigger NETosis by exposed β-1,3-glucans on the surface of the fungal cell wall in comparison to immunologically inert structures that are typical of conidia [27]. NETs, which can be observed *in vitro* and *in vivo*, exhibit mainly fungistatic effects in *A. fumigatus.* Further fungicidal effects have been reported for both yeast and hyphal forms of *C. albicans* [120]. NETosis has also been observed in response to *Cryptococcus neoformans* [121] and *Paracoccidioides brasiliensis* [122].

### 4.3. Proteome Analysis of Neutrophil-Pathogen Interactions

To elucidate the host-pathogen interplay, only a few dual transcriptome and proteome studies have been performed for a better understanding of fungal immune evasion strategies and the screening of promising candidates for novel therapeutic targets [28,34,123]. Such studies can represent a challenge for a number of reasons. Using equal cell ratios (neutrophil *versus* fungal cell count), the higher neutrophil cell size usually results in a higher neutrophil proteome coverage compared to the fungal proteome. In addition, the different levels, on which neutrophils can interact with fungi (intracellular or extracellular) offer different possibilities for performing proteome studies. This includes investigating the phagolysosome proteome and intracellular survival of conidia, identifying released antimicrobial effector molecules against different morphotypes of the fungus in the secretome, and proteome characterization of neutrophil extracellular traps induced by fungal pathogens. Depending on the aim of the study, different approaches should be undertaken. If the underlying goal is to understand the interference of the pathogen with phagolysosome formation and maturation, magnetic bead separation or fluorescence-activated cell sorting (FACS) of labeled pathogen-containing phagolysosomes would be the best choice.

Upon phagocytosis, neutrophils have extracellular killing strategies involving the release of reactive oxygen species and the secretion of antimicrobial effector molecules [12,116,124]. Fungal counter-defense strategies include secretion of proteases or extracellular vesicles. As a consequence, secretome analysis can be very fruitful in studying the host-pathogen interaction that is related to extracellular proteins involved in the defense and counter-defense strategies. The classical approach to purify and enrich secreted extracellular proteins relies most often on the trichloroacetic acid (TCA) protein precipitation (combined with acetone washing steps) of the cell-free culture supernatant or filtrate [125]. Proteins can also be concentrated and enriched by application of molecular weight cut-off filtration techniques [126]. In case of low protein concentrations or proteins that poorly precipitate, the enrichment of proteins based on solid-phase extraction using C4 or ion exchange resins can be the method of choice. These enrichment strategies, however, are unable to distinguish between secreted proteins and proteins released by lysis of dead cells during the cultivation process. This is an important issue due to the low abundance of secreted proteins in comparison to the background noise of intracellular proteins accidentally released into the medium. To some extent, cultivation of leukocytes and maintenance of their vitality highly depends on the addition of high abundant stabilizing proteins based on albumins or fetal calf serum (FCS). This makes it often unfeasible to perform a secretome analysis covering low abundance secreted proteins without depletion of those abundant protein-based culture additives. To overcome this pitfall, Eichelbaum *et al.* [127] developed a click chemistry approach to analyze time-resolved changes of the secretome in response to certain stimuli. In order to capture newly synthesized proteins that were secreted after the onset of cellular stimulation, the authors labeled cells with azidohomoalanine, an azide analog of methionine, to capture newly synthesized secretory proteins by an alkyne-activated resin. This method can be combined with quantitative proteomics techniques.

NET formation has been an object of interest of many *in vivo* and *in vitro* studies towards understanding their function [27,28]. For positive controls, chemical NET inducers such as phorbol myristate acetate (PMA), fMLP, IL-8, C5a, β-1,3 glucans can be used to investigate proteome differences during NET induction by the fungal pathogen in comparison to chemical inducers. The choice of such chemical inducers influences the composition of the NETs [128]. Since the highly abundant NETotic DNA interferes with the protein extraction procedure, nuclease treatment is important for NET proteome analysis. Only one exclusive study by Urban *et al.* [28] focused on the quantitative proteome of NETs induced by PMA and NETs composition induced by *C. albicans* [28]. By means of the nano LC-MALDI-MS analysis, the authors identified 24 NET-associated proteins of nuclear (core histones and myeloid cell nuclear differentiating antigen, MNDA), granular (α-defensins, azurocidin and lysozyme C), and cytoplasmatic origin (glycolytic enzymes, catalase, cytoskeletal proteins and S100 proteins). Normalization for the quantitative approach based on immunoblotting was based on DNA/protein ratios. Interestingly, the four abundant core histones showed changed stoichiometric ratios in PMA induced NETs *versus* resting neutrophils. It has been further shown that NETs triggered by *C. albicans* contained a calprotectin complex consisting of S100A8 and S100A9 that is essential for antifungal defense [28]. O’Donoghue *et al.* [129] characterized the proteolytic activities during PMA-induced NETosis. Based on a peptide library and a fluorogenic substrate library, the activity and cleavage site specificity could be profiled in detail. The proteolytic landscape obtained with the multiplex substrate profiling-mass spectrometry showed that neutrophil elastase (NE) contributed to cleaving 70% of the peptide sites, while 25% of cleavage sites are originated from activity of cathepsin G and only 5% of proteinase 3 (PR3). In addition, the neutrophil serine protease 4 (NSP4) was first identified as NET-associated enzyme [129].

### 4.4. Studying the Phosphoproteome of Neutrophils: Protein Extraction Requirements

Dynamic changes in the post-translational modified protein phosphorylation state of neutrophils have a major impact on the regulation of these immune effector cells in response to certain pathogens. The neutrophil proteome has been analyzed in several studies [119,130,131,132,133,134]. Interestingly, tyrosine phosphorylation modulates crucial neutrophil cell effector functions such as ROS generation via the NADPH oxidase complex, chemotaxis, and integrin-assisted adherence, as well as phospholipase D activation [135]. Preservation of the protein tyrosine phosphorylation status of neutrophil lysates is conceptually and experimentally challenging. Tyrosine phosphorylation is a transient dynamic process that is very rare in comparison to serine/threonine phosphorylation sites. Neutrophils are armed with a plethora of phosphatases and proteases that are activated and hard to control under *ex vivo* conditions unless a wide range of inhibitor cocktails is applied to the sample [135,136]. Furthermore, the phosphorylation level of proteins can be artifactually increased by the presence of Mg^2+^ (tyrosine kinase co-factor), which is sometimes used to preserve the cytoskeletal and membrane integrity [136]. Solubility and extractability of tyrosine-phosphorylated proteins have been shown to be highly dependent on the detergent used in the lysis buffer, since phosphoproteins can form multimolecular complexes in the detergent-insoluble fraction [137].

## 5. Challenges in the Simultaneous Dual Proteome Analysis of Host-Pathogen Interactions

Proteomics is a promising tool to investigate molecular mechanisms in host-pathogen interactions and their dynamics in the course of fungal infections. Yet the enthusiasm in the early postgenomic era changed to a more realistic assessment, since the most promising approaches are often the most ambitious ones. In the following section, we discuss analytical challenges and strategies in the context of proteome analysis dealing with the interaction of host immune cells and various fungal pathogen morphotypes (Figure 1).

### 5.1. Protein Complexity

A major ambition of proteome research is to cover thousands of proteins with diverse functional and physicochemical properties. Fungal and human genomes can encode up to 10,000 [138,139,140] and more than 20,000 proteins [141], respectively. Although the transcription of numerous genes is conditionally silenced, proteome research has to deal with millions of proteins from a chemical perspective and due to alternative splicing and numerous post-translational modifications, (PTMs) that changes the structure, folding, and functionality of the translated proteins. When studying host-pathogen interactions with regard to the response of both the host and the pathogen proteome, the enormous complexity of the sample is almost doubled. This is particularly demanding if one considers that bottom-up proteomic approaches, in which proteins are identified based on specific protease-digested peptides, are currently still the most popular and promising techniques. When analyzing the host and the pathogen proteomes of direct interacting cells simultaneously, the complexity of the digested peptides needs to be reduced by sophisticated (multidimensional) separation and fractionation techniques to handle the vast complexity of the samples and to cover low abundance proteins that might be potential targets or biomarkers of the host-pathogen interaction.

Extensive pre-fractionation procedures such as the multidimensional protein identification technology (MudPIT) analysis can be a powerful tool to manage the overwhelming sample complexity. MudPIT combines different liquid chromatography separation principles based on the molecular weight (size exclusion chromatography), the isoelectric point (cation/anion exchange), the hydrophobicity (reversed phase, mixed mode or ion pairing chromatography), the hydrophilicity (hydrophilic interaction (HILIC) or electrostatic repulsion (ERLIC)). Further fractionation and enrichment strategies rely on modifications such as glycosylation (lectin affinity) and phosphorylation (TiO_2_, IMAC, SCX, ERLIC). The enrichment of other PTMs (e.g., acetylation, ubiquitination) is usually performed on the peptide level based on specific antibodies (e.g., anti-acetyl or anti-diglycyl lysine antibodies). Furthermore, the online liquid chromatography separation performance at nano flow rates has been massively improved over the years using ultralong gradients [142] and/or non-linear gradients for an evenly distributed peptide elution profile [143]. Finally, improvements in the resolution and scan speed of state-of-the-art mass spectrometry instruments consistently enhance the overall proteome coverage of in-depth analyses. The increasing comprehensiveness of proteomics datasets makes it necessary to pay more attention to the downstream analysis in order to extract biological meaning out of the data. Several tools are available that help to interpret the protein database search results by means of gene ontology functional annotation, pathway analysis, and prediction of signal peptides and interaction networks [144,145,146,147,148]. In addition, Perseus/MaxQuant [149], the OpenMS proteomics platform [150], and PeptideShaker [151] are commendable and freely available tools to extract and visualize biologically meaningful information from shotgun proteomics data.

### 5.2. Separation of Host Immune Cells from Fungal Pathogen Cells

The application of cell sorting techniques (e.g., FACS) to separate host and pathogen cells prior to the analysis is feasible to circumvent complexity issues caused by simultaneous dual proteome analyses. Such techniques are regularly applied for host cells interacting with bacterial pathogens [152,153]. In contrast, flow cytometry cell sorting techniques of filamentous fungi are not generally advisable due to the cell size and the morphologic heterogeneity of hyphal cells. When neutrophils undergo NETosis as a response to co-incubated fungal cells, they cannot be separated from the pathogen by means of FACS or magnetic cell sorting due to the spreading of networks of intracellular proteins and DNA in their immediate extracellular surroundings. The same applies to phagolysosomes. Separation of the ingested fungal cells from the surrounding host environment is not feasible based on cytometry techniques. Due to the high resistance of most fungal cells against osmotic stress in comparison to highly sensitive phagocytes, host cells can often be lysed (depending on the experimental setup) and thus separated from the fungal pathogen by using deionized water. Indeed, separation of proteins from both origins remains impossible for secretome analyses. The simultaneous dual proteome analysis remains the method of choice when cell sorting techniques are not applicable.

### 5.3. Dynamic Range of Dual Proteome Studies

The dynamic range of protein abundance in eukaryotic cells is up to six orders of magnitude [154]. For body fluids with high albumin content such as blood plasma the dynamic range can cover even up to 10 orders of magnitude [155]. Dual proteome studies analyzing the host and the pathogen proteome simultaneously (e.g., phagocytosed fungal cells) make analyses even more complicated, since the dynamic range is influenced by both the average copy number of each protein in the cell and the quantitative ratio of both proteomes contributing to the whole protein sample. This ratio depends on the multiplicity of infection (MOI), *i.e.*, the quantitative ratio of phagocytic immune cells interacting with a defined number of fungal cells, and the average total protein concentration per cell type. Since fungal cells can be morphologically diverse, the proteome ratio can highly differ, depending on the experimental setup, the organism, and its morphotype (yeast, conidia, hyphae, *etc.*). As a consequence, it is recommended to find a balanced compromise for the experimental setup. On one hand, a realistic and less artificial MOI ratio should be applied. On the other hand, the quantitative ratio of the total protein amount of the host cell in relation to total protein amount of the pathogen should not differ hugely from a proportion of 1:1. Otherwise, the dynamic range would be increased leading to a downgrading of the overall output of the relative quantitative analysis.

### 5.4. Quantitative Proteomics

The impact of the data quality of such in-depth proteome analysis has been widely underestimated in terms of quantitative proteomics strategies. It has been demonstrated that the majority of identified peptides of in-depth proteome analysis has signal/noise (S/N) ratios of less than 10, with over a quarter of all peptides identified at an S/N ratio of less than 5. While such low signal levels do not necessarily affect the quality of the MS/MS spectra in terms of the peptide identification rate, this circumstance has a significant negative influence on the accuracy and precision of quantitative calculations. The accuracy of any relative quantitative measurement is highly correlated with the S/N ratios [156]. Moreover, peptide recovery issues after extensive offline fractionation procedures using the MudPIT approach can be a striking pitfall due to a significant sample loss and error propagation in the course of sample preparation procedure [157,158]. In this context, strategies for the quality control should be considered with regard to the reliability and reproducibility of the applied sample preparation procedure [159].

Several methods have been applied to quantify shotgun proteomics data. Label-free quantification methods are mainly based on spectral counting or area under the curve calculations [140]. The number of peptide spectrum matches (PSMs) used for spectral counting highly depends on the complexity and dynamic concentration range of proteome samples. In dual proteome analysis, where host-pathogen interactions are compared against separate host and pathogen controls, spectral counting necessarily leads to unacceptable variations. Area under the curve approaches comparing the peak intensity/area of precursor ions among different sample groups are theoretically more reliable, but often highly dependent on the alignment of the retention time of relatively quantified precursor ions and the robustness and reproducibility of the LC-MS/MS analysis. The number of replicates needs to be high enough to generate statistically solid data. This can be restrictive in terms of host immune cells isolated from limited volumes of blood samples. Label-based quantification methods are often more reliable and allow multiplexing. Metabolic labeling procedures such as stable isotope labeling by amino acids in cell culture (SILAC) have the advantage that the proteins are labeled during protein biosynthesis. However, the labeling efficiency by metabolic incorporation of light/heavy amino acids is often critical, especially when working with short-lived neutrophils and filamentous fungi, which require the generation of arginine and/or lysine auxotrophic mutants. In addition, chemical labeling techniques are available, including isotope-coded protein labeling for MS1 quantification (ICPL, ICAT) and isobaric tag peptide labeling for MS2 reporter ion quantification (iTRAQ, TMT). The latter can be multiplexed up to 10-plex. Isolation of the precursor ions undergoing MS2 fragmentation can result in ratio distortion effects if nearly isobaric peptides are co-isolated. This effect can be reduced by offline pre-fractionation, narrow isolation widths, and most effectively by MS3 scans [160].

### 5.5. Normalization of Quantitative Dual Proteome Analysis

**Figure 2:**
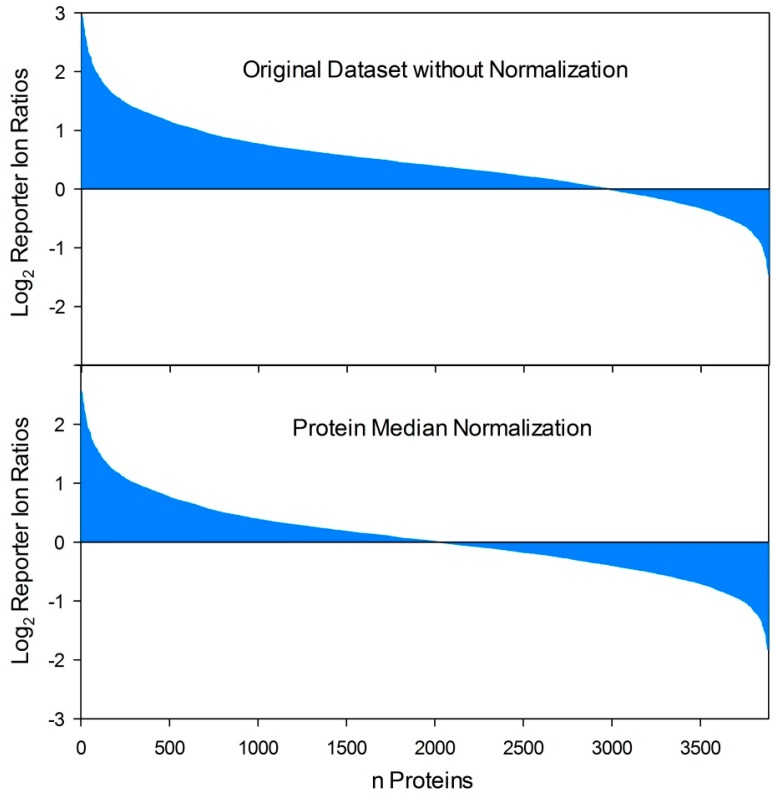
Effects of protein normalization procedures on quantitative proteome datasets. By comparison of two proteome conditions (e.g., host-pathogen interaction versus control) based on reporter ion quantification procedures (iTRAQ, TMT) the respective ratio calculation resulted in an uneven distribution of protein fold changes if varying total protein concentrations were compared. In this example, this results in several false positive “upregulated” reporter ion ratios while the ratios of moderate down-regulated proteins (depending on the applied threshold value for differential regulation) are dropped under the threshold value as false negatives. In contrast, protein median normalization resulted in an even distribution of protein fold changes and is capable to equalize disproportionate samples and allow comparability in a relatively quantitative manner.

The most important issue related to dual proteome analysis is the choice of an appropriate method for normalization of the initial protein amounts used for relative quantitative analysis. When analyzing single proteome samples based on quantitative proteomics techniques, it is necessary to compare an equally defined total protein quantity of all comparison groups (e.g., mutant *vs.* wild type, treatment *vs.* control, different time points, *etc.*). Replicates to ensure that experimental observations of up- or downregulated protein levels of certain proteins are also important due to the differential expression of the corresponding protein and not the result of different initial total protein amounts. Consequently the precision and robustness of the total protein quantification method is an important issue. To some extent deviations based on measurement errors can be normalized *in silico*, for instance, based on a protein median normalization (Figure 2). This approach has also been applied for the first report of a mixed and quantitative dual proteome analysis that has been recently published based on the interaction of *C. albicans* with murine macrophages. The authors identified 483 *C. albicans* and 1253 macrophage proteins in total, where of 227 *C. albicans* and five macrophage proteins of the co-culture experiment exhibited a differential expression compared to separate *C. albicans* and macrophage mono-culture controls [161]. Reporter ion quantification using the TMT labeling approach was applied to reveal relative quantitative changes on the proteome level [161]. The protein median normalization approach cannot be recommended for all types of experimental setups and outcomes, in particular when a certain response on the actual protein level shows a significant but moderate differential regulation (e.g., 2.5-fold up) of numerous proteins in one direction and a drastic differential regulation (e.g., 25-fold down) of few proteins in the other direction. Protein median normalization would drop numerous interesting actual upregulated proteins under the significance threshold. Several alternative *in silico* normalization approaches were compared in a recent publication [162]. The most promising method was the invariant marker set concept. This approach depends on the identification of a set of non-regulated proteins that serve as a virtual reference used for normalization [162].

In dual proteome analysis of host-pathogen interactions that do not allow separation of the host immune cells from the fungal pathogen, protein normalization should not be entirely left to *in silico* methods. Normalization based on a sophisticated experimental setup is way more challenging. For this purpose, it is necessary to evaluate the proportion of both the host and the pathogen proteome contributing to the total protein amount measured after protein isolation. As host-pathogen interaction studies are usually based on co-incubation experiments, investigators are often not able to determine the corresponding proportion at the endpoint of the incubation. The quantitative proportion of the host proteome in comparison to the pathogen can be determined at the starting point of the incubation on the eve of the inoculation of the host immune cells with the fungal cells. Proper homogenization of the cell suspensions without disturbance of the activity and viability of the cells is an important prerequisite for quantitative experiments. Such pre-incubation normalization approaches can be error-prone depending on the duration of the co-incubation experiment. On one hand, investigators aim to observe distinct proteomic responses of the host-pathogen interaction on both sides. On the other hand, changes on the protein level in the course of the co-incubation experiment complicate an exact normalization. Theoretically, the major influence on the overall protein amount during co-incubation experiments could be attributed to the growth (cell proliferation and division) of the fungal pathogen. Since host immune cells such as neutrophils and macrophages are very sensitive in terms of their viability and activity, the medium used for co-incubation experiments is mostly optimized for the requirements of the host immune cells, resulting in sub-optimal growth rates of the fungal pathogen. Additionally, host-pathogen interaction is obviously a stress condition that does not necessarily favor growth of the fungal pathogen. To a limited extent, the viability of the immune cells and the growth of the fungus during the co-incubation experiment can be observed based on separate controls with either host immune cells or fungal cells incubated separately in the same medium used for the co-incubation experiment. Finally, there is a strong need for further developments on the quality control and normalization of dual proteome analysis to differentiate the host and the pathogen proteome in a quantitative manner.

## 6. Conclusions

Proteome analyses of the interaction of human pathogenic fungi with host immunity have the potential to give insights into the molecular interplay of host immune defense mechanisms, the immune evasion strategy and stress response of fungal pathogens. The interplay of the adaptive immunity with fungal proteins is the basic subject of immunoproteomic techniques dealing with the identification of antigenic proteins that may serve as diagnostic biomarkers and targets for vaccination and therapeutic interventions. Further dual proteome studies on the direct interaction of phagocytic immune effector cells with human-pathogenic fungi are challenging for a number of reasons, including the selection of blood donors, the demands on isolation procedures and culture conditions for immune effector cells, the selection of an appropriate MOI, the homogeneity of the cell suspensions used for inoculation, the demands on normalization strategies, and the enormous sample complexity including expanded dynamic ranges. Numerous strategies exist to overcome these obstacles, although a sophisticated experimental design is crucial to obtain reliable results. In this context, investigators always have to compromise between conditions that are not too artificial to mimic realistic conditions and that keep, at the same time, the experiment controllable and the outcome reproducible. Proteome research on host-pathogen interactions with respect to fungal pathogens is still at its infancy. Extensive effort in future research is required to obtain deeper insights into the mechanisms of pathogenicity and virulence.
